# Therapeutic Effect and Mechanism of Si-Miao-Yong-An-Tang on Thromboangiitis Obliterans Based on the Urine Metabolomics Approach

**DOI:** 10.3389/fphar.2022.827733

**Published:** 2022-02-22

**Authors:** Hui-Yu Li, Hui Sun, Ai-Hua Zhang, Lu-Wen He, Shi Qiu, Jun-Ru Xue, Fangfang Wu, Xi-Jun Wang

**Affiliations:** ^1^ National Engineering Laboratory for the Development of Southwestern Endangered Medicinal Materials, Guangxi Botanical Garden of Medicinal Plant, Nanning, China; ^2^ National Chinmedomics Research Center, National TCM Key Laboratory of Serum Pharmacochemistry, Functional Metabolomics Laboratory, Heilongjiang University of Chinese Medicine, Harbin, China; ^3^ State Key Laboratory of Quality Research in Chinese Medicine, Macau University of Science and Technology, Macao SAR, China

**Keywords:** metabolomics, biomarker, therapeutic effect and mechanism, pathway, metabolites

## Abstract

Si-Miao-Yong-An-Tang (SMYAT) is a classic prescription for the treatment of thromboangiitis obliterans (TAO). However, the effect and mechanism are still unclear. This experiment aims to evaluate the therapeutic effect and mechanism of SMYAT on sodium laurate solution induced thromboangiitis obliterans model rats using urine metabolomics. The therapeutic effect of SMYAT was evaluated by histopathology, hemorheology and other indexes. The urine metabolomic method, principal component analysis (PCA) and orthogonal partial least squares discriminant analysis (OPLS-DA) were used for clustering group and discriminant analysis to screen urine differential metabolic biomarkers, and explore new insight into pathophysiological mechanisms of SMYAT in the treatment of TAO. SMYAT has significant antithrombotic and anti-inflammatory effects, according to the results of urine metabolomic analysis, and regulate the metabolic profile of TAO rats, and its return profile is close to the state of control group. Through metabolomics technology, a total of 35 urine biomarkers of TAO model were characterized. Among them, SMYAT treatment can regulate 22 core biomarkers, such as normetanephrine and 4-pyridoxic acid. It is found that the therapeutic effect of SMYAT is closely related to the tyrosine metabolism, vitamin B6 metabolism and cysteine and methionine metabolism. It preliminarily explored the therapeutic mechanism of SMYAT, and provided a scientific basis for the application of SMYAT.

## Introduction

Traditional Chinese medicine (TCM) is widely used in China, and its curative effect is remarkable (Wang et al., 2011; Zhang et al., 2013; Guo et al., 2020). However, due to the lack of modern scientific evidence to prove the use of TCM, the mechanism of action is unclear, leading to the therapeutic effect of TCM did not play an original potential. Metabolomics is an innovative method in modern research and it can find biomarkers and clarify biochemical pathways, thereby improving diagnosis and treatment (Zhang et al., 2012a; Liang et al., 2015a; Liang et al., 2016b). The analysis of key biomarkers and the monitoring of metabolic pathways are, in analytical biology, an important means of physical state (Zhang et al., 2012b). Its purpose is to measure a wide range of small molecules under physiological stimulation or disease state (Roberts et al., 2012; Filla and Edwards, 2016). Due to the high sensitivity of metabolomics, it can detect subtle changes in metabolic pathways to explore the underlying mechanism of disease (Johnson et al., 2016). At present, metabolomics has been increasingly used to identify biomarkers in diseases (Liang et al., 2014; Liang et al., 2015b), and is considered a very powerful tool.

Metabolomics analysis based on mass spectrometry provides information on the metabolic pathways in the development of thromboangiitis obliterans disease, which has a certain reference value for studying the potential mechanisms of treatment strategies. Metabolomics can provide metabolic fingerprints of organisms, which can be used to study the mechanism of disease (Zhang et al., 2015; Zhang et al., 2019 A.-H.). It can explore metabolites and body pathology through high-throughput analysis of metabolites in organisms and explore the correlation between metabolites and the pathophysiological changes of the body (Li et al., 2018; Zhang et al., 2016; Zhang et al., 2019 AH.), and clarifies the interaction of complex systems in the body (Liang et al., 2016a; Liang et al., 2017; Zhang et al., 2018). It can btain corresponding biomarkers through metabolomics technology to characterize or reveal the functional state of a specific time or environment (Zhang et al., 2017; 2020). For example, by establishing a powerful metabolomics strategy to explain the pathological changes of Yanghuang syndrome and the therapeutic effect of TCM, and the Yin-Chen-Hao-Tang was used to achieve significant results (Liu X.-y. et al., 2018). Through metabolomics, the mechanism of Gancao Fuzi decoction achieved good results in the treatment of rheumatoid arthritis in rats by regulating various metabolic pathways (Liu Y. et al., 2018). Using the metabolomics method, the effect of *Angelica sinensis* on urinary metabolites in blood deficiency in mice was studied and the mechanism of blood tonifying was explored. It was found that *Angelica sinensis* could regulate a variety of metabolic pathways and exert a curative effect (Wang et al., 2016). In our research, the potential mechanism of SMYAT in the treatment of thromboangiitis obliterans through multi-component, multi-target, and multi-channel regulation of body function is explored by metabolomics. The study of metabolomics and related pathways will help us to increase our understanding of pathophysiology and mechanisms of disease (Laíns et al., 2019). Moreover, urine is one of the important samples for metabolome research (Zhang et al., 2012c; Harpole et al., 2016), which contains a variety of endogenous small molecule metabolites and can well reflect the changes of the body. As a very attractive sample in metabolomics research, it has several advantages, such as low protein content and simple pretreatment (Khamis et al., 2017).

Thromboangiitis obliterans (TAO) is a staged inflammatory disease and mainly occurs in the arteries, veins, and nerve parts of the limbs (Quintas and Albuquerque, 2008; Piazza and Creager, 2010; Highlander et al., 2011). The clinical symptoms are mainly manifested in the following conditions: limb ischemia; cold; severe pain limp, weakening or disappearance of arterial pulsation; gangrene, etc. Serious cases can endanger limbs and life and . the exact pathogenesis and etiology of the disease are not clear (Fazeli and Ravari, 2015; Li et al., 2020). In this experiment, a rat model of thromboangiitis obliterans was induced by an injection of sodium laurate solution. Sodium laurate has a strong endothelial damage effect and can cause the vascular endothelium to fall off. It is the most commonly used method of modeling (Munnix et al., 2009; Asada et al., 2018; Liu et al., 2019; Wang et al., 2021). Existing models mostly evaluate the success of model preparation by observing changes in apparent behavior and the degree of gangrene, hemorheology, and histopathology are the most commonly used detection indicators. In our research, hemorheology and histopathology test results support the successful model building. The SMYAT is included in《YAN-FANG-XIN-BIAN》 (Li et al., 2013; Yang et al., 2020), and is a famous classic prescription in ancient China, which is composed of four kinds of traditional Chinese medicines, namely Flos *Lonicerae Japonicae* [*Lonicera japonica* Thunb. (Jinyinhua), Caprifoliaceae], Radix *Scrophulariae Ningpoensis* [*Scrophularia ningpoensis* Hemsl. (Xuanshen) Scrophulariaceae], Radix *Scrophulariae Ningpoensis* [*Angelica sinensis* (Oliv.) Diels (Danggui) Apiaceae], and Radix *Glycyrrhizae Uralensis* [*Glycyrrhiza uralensis* Fisch. (Gancao) Leguminosae]*.* (Peng et al., 2012; Liu et al., 2017; Zhong et al., 2017), which contain terpenoids, flavonoids, phenylpropanoid, and organic acids, which can achieve anti-inflammatory, anti-oxidation stress, regulating blood lipid, inhibiting thrombosis and improving hemorheology, etc. (Zheng et al., 2019). It has the function of clearing heat and detoxifying poison. It is commonly used in the treatment of thromboangitis obliterans (Zhao et al., 2020). In our experiment, we analyzed the chemical components in the sample of Si-Miao-Yong-An-Tang, and a total of 79 chemical components were characterized. The relevant chromatograms and specific information tables of the compounds are shown in Supplementary Material S1. This study determined urine biomarkers of TAO model rats, which were used to distinguish between the control group and the TAO model groupThe biomarkers are expected to be used as therapeutic targets and to regulate relevant monitoring and treatment biomarkers when using SMYAT.

## Materials and Methods

### Instrument

Acquity™ UPLC liquid chromatography (Waters, USA); Synapt™ G2-Si mass spectrometry (Waters, USA); Masslynx v4.1 workstation (Waters, USA); Ultrasonic cleaner (KQ-250 DB, Kunshan Ultrasonic Instrument Co., Ltd., China); Table centrifuge (sorvall ST 16R, Thermo Scientific, USA); Thermo Scientific 995 ultra low temperature refrigerator (Thermo Scientific, USA).

### Reagents and Materials

All herbs were obtained from Harbin No.4 Chinese Medicine Factory Co., Ltd. Flos *Lonicerae Japonicae* (Lot number: JL030-180401), Radix *Scrophulariae Ningpoensis* (Lot number: JL031-180601), Radix *Angelicae Sinensis* (Lot number: JL048-180601) and Radix *Glycyrrhizae Uralensis* (Lot number: JL054-180501) were weighed out according to the ratio 3:3:2:1 and soaked in distilled water for 30 min and boiled for 1 h. The solution was filtered and freeze-dried under vacuum to obtain a loose powder and freeze-dried powder was dissolved in water and prepared in 8.2 g/kg solution for oral gavage administration to rats. In addition, we carried out quality control on the sample of Si-Miao-Yong-An-Tang. For details, please refer to the Supplementary Material S2.

Mai-Luo-Ning Granules (MLN) are provided by Jiangxi Yintao Pharmaceutical Co., Ltd. (Lot number:1808025). Sodium Laurate are provided by Sinopharm Chemical Reagent Co., Ltd. (Lot number:20171224). Methanol and acetonitrile (HPLC grade) were purchased from Fisher Scientific Corporation (Fisher, United States); leucine enkephalin was purchased from Sigma-Aldrich (SIGMA, United States); formic acid (HPLC grade) was purchased from Aladdin Industrial Corporation (Shanghai, China); sodium chloride injection was purchased from Kelun Pharmaceutical Co., Ltd., (Sichuan, China); all other reagents were HPLC grade.

### Animal Models and Study Design

Sixty male SD rats with the weight of (280–320) g were purchased from the Liaoning Changsheng Biotechnology Co., Ltd. [Permit number: SCXK (Liao) 2015-0001]. Those rats were exposed to a temperature of 24 ± 2°C and the humidity of 40 ± 5%, the animals were reared in separate cages, all rats were adaptive fed for 7 days and then randomly divided into four groups: Control group (*n* = 20), Model group (*n* = 20), SMYAT group (*n* = 10), and MLN group (*n* = 10). Rats in model group, SMYAT group and MLN group were injected with a sodium laurate solution to induce thromboangiitis obliterans model, while rats in control group were injected with the same amount of normal saline. After 10 days, the rats in SMYAT group (rats were fed with SMYAT 8.2 g/kg per day for 15 days) and MLN group (rats were fed with MLN 2.7 g/kg per day for 15 days) were given medicine treatment. The rats in control group and thromboangiitis obliterans model group were given pure water. This research was approved by the Ethical Committee of Heilongjiang University of Chinese Medicine and was conducted according to the principles expressed in the Declaration of Helsinki.

### Sample Preparation

#### Urine Sample Preparation

Control group and model group of urine samples were collected at 10th day of the experiment. Accompanying control group, accompanying model group, SMYAT group and MLN group of urine samples were collected at 25th day of experiment and then centrifuged with of 13,000 rpm/min in 4°C for 10 min to obtain urine samples. Before UPLC-MS analysis, the urine supernatant was stored at −80°C.

Before analysis, the urine samples were thawed in an ice bath, 500 μL of urine was taken and added to 500 μL of ultra-pure water, placed in a vortex mixer for 30 s, centrifuged at 13,000 rpm for 15 min at 4°C; and filtered over 0.22 μm, then the supernatant were injected into the UPLC-MS.

#### Pharmacodynamic Evaluation Sample Preparation

The accompanying control group, accompanying model group, SMYAT group and MLN group of blood samples were collected from the abdominal aorta of rats (on the 25th day of the experiment, 1 h after the last administration). Sodium citrate and EDTA-K2 were used for anticoagulation. The whole blood viscosity and plasma viscosity and other indexes of rats in each group were determined. In each group, the femoral artery tissues below 2–3 cm and around the artery were taken from the affected limb. After washing with normal saline, the femoral artery was fixed with 10% formalin solution for 24 h for histopathological observation.

### Analysis of Samples by HPLC-MS

#### Chromatographic Conditions

Chromatograph condition: Acquity™ UPLC liquid chromatography (Waters, USA); chromatographic column used was an ACQUITY HSS T3 chromatographic column (100 mm × 2.1 mm i.d., 1.8 μm. Waters, USA); the column temperature is set at 40°C; the sample bin temperature is set at 4°C; meanwhile mobile phase for gradient elution consist of phase A (acetonitrile containing 0.1% formic acid) and phase B (water with 0.1% formic acid). The gradient was as follows: 0–1 min, 1%A; 1–6 min, 1–30%A; 6–8 min, 30–70%A; 8–9 min, 70–99%A; 9–10 min, 99%A; 10–10.1 min, 99–1%A; 10.1–14 min, 1%A. In addition, equal volume of each sample was mixed into a quality control sample (QC) for monitoring the stability of the instrument in the experiment, and one QC solution was run every ten samples, which were crucial for optimization during metabolomic analytical method development.

#### Mass Spectrometry Conditions

Synapt TM G2-Si mass spectrometer (Waters, USA). MS system using ESI as an ion source to operate in ESI+ and ESI-modes with a scanning range of 50–1,200 m/z. The desolvation temperature was 400°C, the source temperature was 110°C, desolvation gas flow was 800 L/h, and cone gas flow was 50 L/h. Sampling cone voltage was maintained at 20 V; Leucine enkaphalin was used as the reference compound {positive ion mode [(M + H)^+^ = 556.2771] and (M−H)^−^ = 554.2615}. All the above parameters are set using Masslynx v4.1 workstation (waters, United States).

### Metabolomics Data Processing

In order to more accurately find the urine biomarkers in TAO model rats original urine files were imported into Progensis QI software for analysis and processing, andpeak matching and normalization for each peak was performed. Meanwhile, we used Ezinfo 3.0 software, including PCA and OPLS-DA. SPSS 22.0 for Windows was used for the statistical analysis. The variable importance for the projection (VIP) value >2 in OPLS-DA, and *p* < 0.05 for Students t-test were selected as potential urine biomarkers.

The identification of biomarkers is to lock the Rt_m/z of important metabolite ions to help confirm the molecular mass of metabolites and perform elemental composition analysis to determine possible chemical formulas. Based on the possible chemical formulas, accurate masses and secondary mass spectrometry data (MS/MS) of ions, the Human Metabolome Database (HMDB) and Metaboanalyst were used to match the mass spectrometry information. The MassFragment application manager was used to facilitate the chemically intelligent peak matching algorithms MS/MS fragment ion analysis process and to determine the final biomarker. The results of the secondary ion fragmentation information matching of these 35 compounds are shown in Supplementary Material S3.

## Results

### Biomarkers Discovery of TAO Model

**FIGURE 1:**
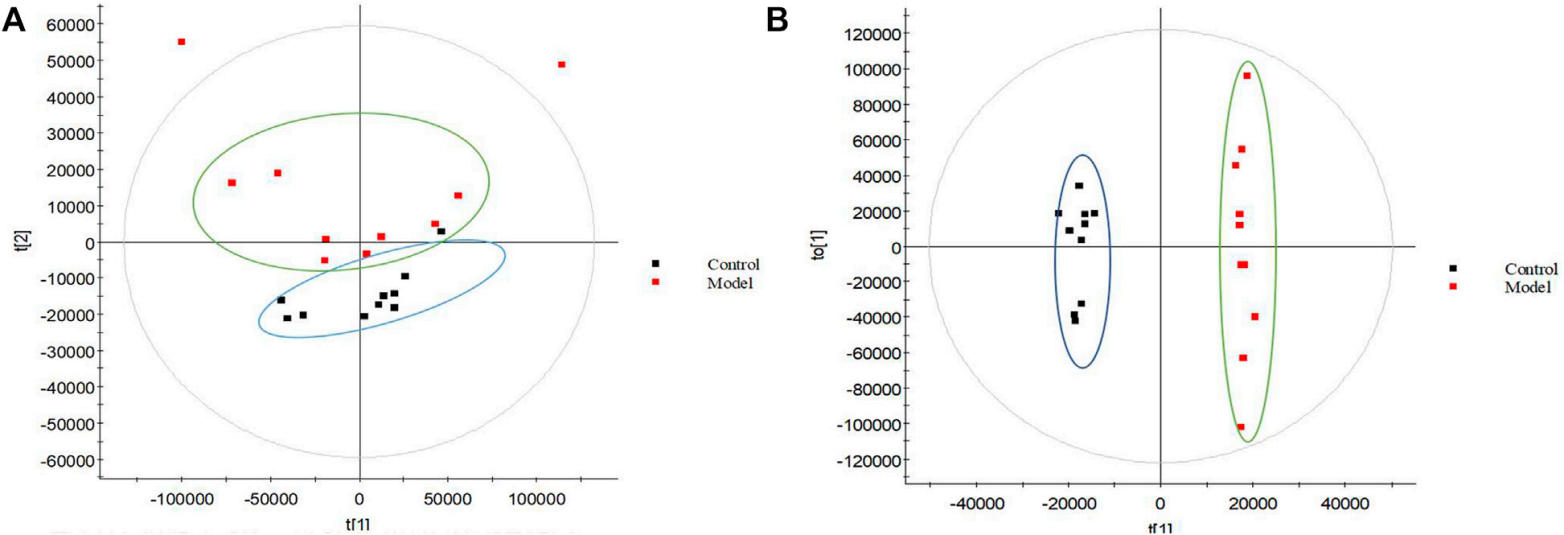
Metabolomic profiling of TAO. PCA model results for TAO model group in positive mode **(A)**; OPLS-DA model results for TAO model group in positive mode **(B)**; Black spot represents the control group and red spot represents the model group.

**FIGURE 2:**
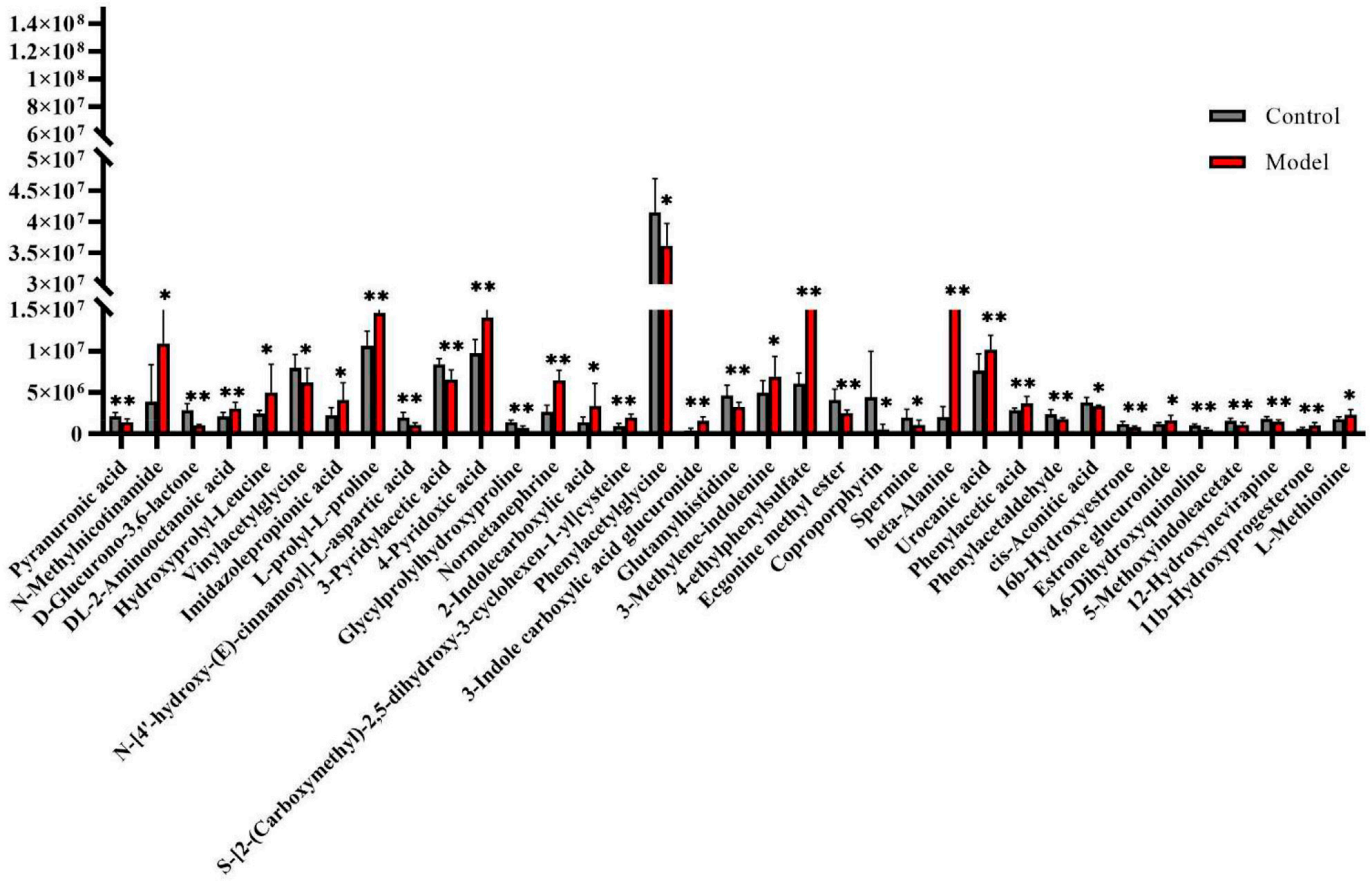
Change trend of biomarker content in control group and model group. *Significant difference between the control group and model group (*p* < 0.05). **Very significant difference between the control group and model group (*p* < 0.01).

**FIGURE 3:**
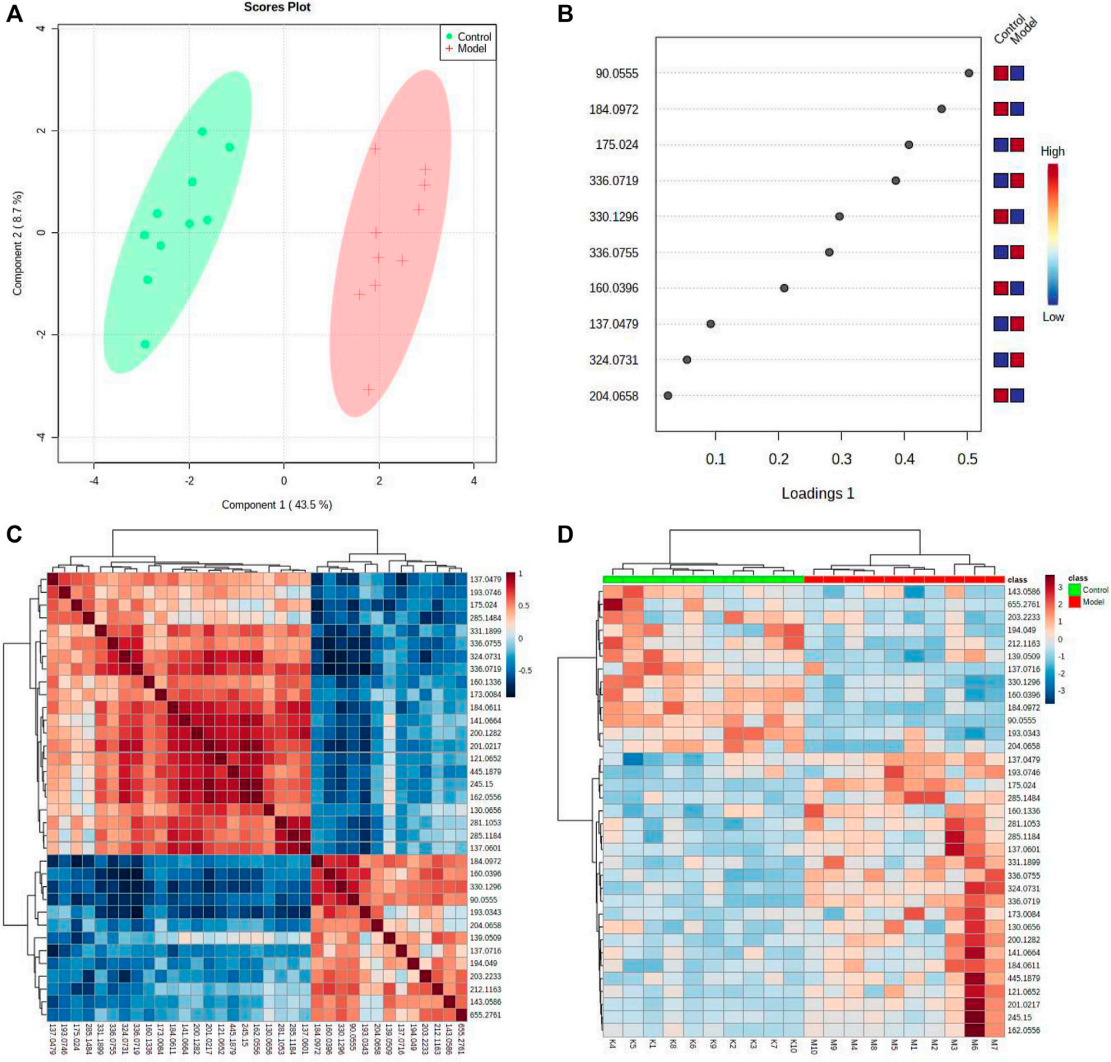
Systems analysis of metabolomic alterations of the model and control samples with MetaboAnalyst’s data annotation tools: **(A)** PLS-plot revealed differences between the two groups. **(B)** Correlation analysis of the 10 differential metabolites are marked on the plot. Three clusters were identified representing the different groups of metabolites. Top significant features of the metabolite markers based the VIP projection **(B)**. **(C)** Hierarchical clustering of the differential metabolites. **(D)** Heatmap visualization constructed based on the differential metabolites of importance for the urine of model. The heatmaps were constructed based on the potential candidates of importance, which were extracted with OPLS-DA analysis. Rows: metabolites; columns: samples; Variable differences are revealed between the control and model groups.

The urine datasets collected on the 10th day were further subjected to multivariate statistical analysis using EZinfo 3.0 software. The PCA score plots (Figure 1A) showed an obvious separation between the TAO model and control model. The OPLS-DA was employed to find the potential biomarkers (Figure 1B). The R2Y and Q2 values of the model [R2Y (cum) = 0.991607, Q2 (cum) = 0.905193 in positive mode and R2Y (cum) = 0.993259, Q2 (cum) = 0.942307] in negative mode indicated that the models have good quality and predictability. The OPLS-DA score plots showed that there was a significant difference between the TAO model groups and control groups, suggesting that the TAO model group had obvious urinary biochemical disorders and its metabolic profile had changed significantly. Screen out potential urine markers, and by using related databases, such as HMDB, KEGG, etc. Meanwhile, we combined the fragment information of the secondary mass spectrometer to obtain the chemical information of potential biomarkers, see Supplementary Table S1 for details. The content of these potential biomarkers in the 10th day model group and the 10th day control group were statistically analyzed (Figure 2). We constructed a PLS-DA model with the obtained differential metabolic markers (Figure 3A). Figure 3B showed top significant features of the metabolite markers based the VIP projection. Correlation analysis of differential metabolites are marked on the hierarchical clustering plot (Figure 3C), and the obtained differential metabolic markers are analyzed by heat mapwith results shown in Figure 3D.

### Metabolic Pathways of Thromboangiitis Obliterans

**FIGURE 4:**
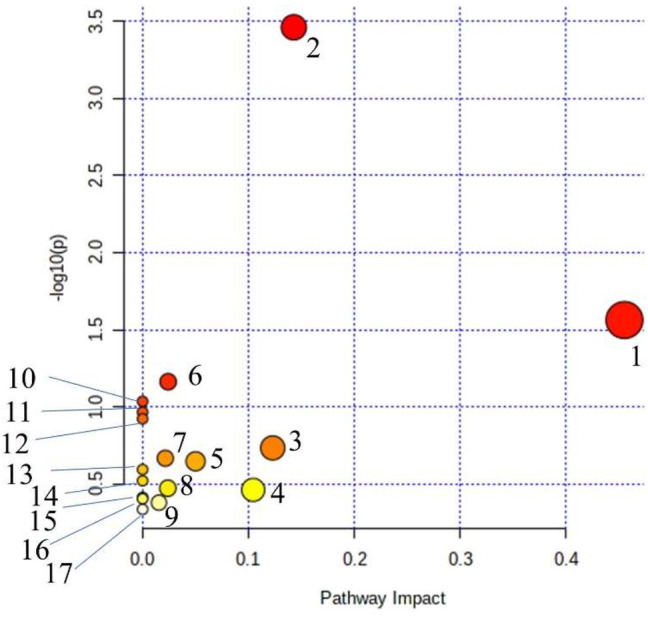
Main metabolic pathways of potential biomarkers. (1) beta-Alanine metabolism; (2) Phenylalanine metabolism; (3) Histidine metabolism; (4) Cysteine and methionine metabolism; (5) Citrate cycle (TCA cycle); (6) Steroid hormone biosynthesis; (7) Pantothenate and CoA biosynthesis; (8) Glyoxylate and dicarboxylate metabolism; (9) Tyrosine metabolism; (10) Tryptophan metabolism; (11) Vitamin B6 metabolism; (12) Ascorbate and aldarate metabolism; (13) Propanoate metabolism; (14) Glutathione metabolism; (15) Arginine and proline metabolism; (16) Pyrimidine metabolism; (17) Aminoacyl-tRNA biosynthesis.

MetPA (Metabolomics Pathway Analysis) is used to analyze metabolic pathways in metabolomics data. MetPA analysis was performed on the characterized 35 biomarkers related to thromboangiitis obliterans model, and 17 related metabolic pathways were obtained including vitamin B6 metabolism, cysteine and methionine metabolism, tyrosine metabolism, phenylalanine metabolism, and so forth. The results show that these endogenous metabolites are closely related to the thromboangiitis obliterans model. The metabolic pathway is shown in Figure 4.

### Efficacy Evaluation of SMYAT

#### SMYAT Improves the Animal Signs of TAO Model Rats

Rats in each group were in good condition, lively and active prior to the start of the experiment. After modeling, except for control group, the other groups of rats showed obvious ischemic symptoms, such as lower skin temperature, obvious swelling of foot and paw, weakened arterial pulsation, limping and dragging walking in varying degrees. On the 10th–25th day, the condition of the affected limbs of the rats in each treatment group was gradually improved, among them, the SMYAT group had a significant effect, the skin temperature returned to normal, the swelling of the paws subsided, and the arterial pulsation recovered, while the state of the rats in the following model group remained the same as that after modeling, and there was no improvement trend.

#### SMYAT Improves the Hemorheology of TAO Model Rats

**FIGURE 5:**
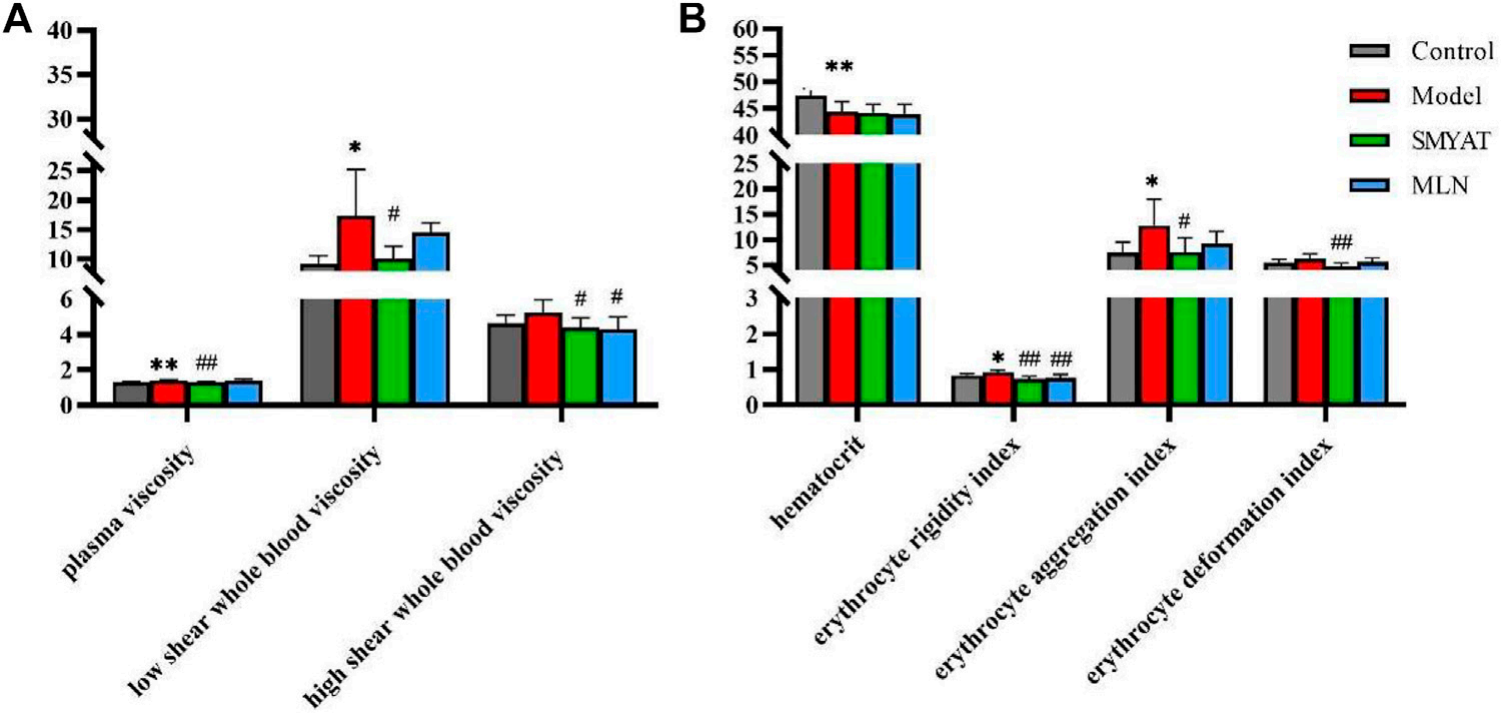
**(A)** Effects of administration on plasma viscosity and whole blood viscosity of rats in each group; **(B)** Effects of administration on related indexes of red blood cells of rats in each group; *p < 0.05, **p < 0.01 compared with control group; ^#^ *p* < 0.05, ^##^ *p* < 0.01 compared with model group.

The results in Figure 5A, show low shear whole blood viscosity and that the blood plasma viscosity of the thromboangiitis obliterans model rats had changed significantly compared with the control group. After treatment with SMYAT, all the indexes had a significant decrease, which showed that SMYAT had a good effect on regulating blood viscosity and treating thrombosis.

The results in Figure 5B, show the hematocrit, erythrocyte aggregation index and erythrocyte rigidity index of the thromboangiitis obliterans model rats changed significantly compared with the control group. After treatment with SMYAT, the erythrocyte rigidity index, erythrocyte aggregation index and erythrocyte deformation index have been significantly adjusted, indicating that SMYAT has a significant regulatory effect on red blood cells and other indicators, and can achieve good therapeutic effects.

#### SMYAT Improves Histopathology of TAO Model Rats

**FIGURE 6:**
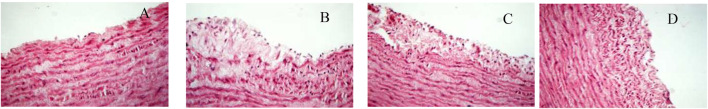
Femoral artery sections of rats in each group on the 25th day of the experiment (×400); **(A)**: control group; **(B)**: model group; **(C)**: SMYAT group; **(D)**: MLN group.

We evaluated the effect of SMYAT on the histopathology of TAO model rats (Figure 6), and observation of rat femoral artery sections showed that there were no pathological changes in the control group, the vascular endothelium was smooth and orderly, and the endothelial cells were neatly arranged (Figure 6A). The femoral artery sections in the TAO model group found obvious pathological changes, such as vascular thinning, endothelial cell deformation, vascular endothelial cells and smooth muscle cells disorderly arranged, and a large number of inflammatory cell infiltration (Figure 6B). In the SMYAT treatment group, the degree of femoral artery lesions was improved, the deformation of endothelial cells was improved, and the arrangement of vascular endothelial cells and smooth muscle cells was restored, and the injure condition was restored and maintained a stable state (Figure 6C). In MLN group, the femoral artery became slightly thinner, intimal peeling phenomenon relieved, and the inflammatory cell infiltration in the blood vessel wall and surrounding tissues gradually reduced (Figure 6D).

### Effect of SMYAT on Biomarkers

**FIGURE 7:**
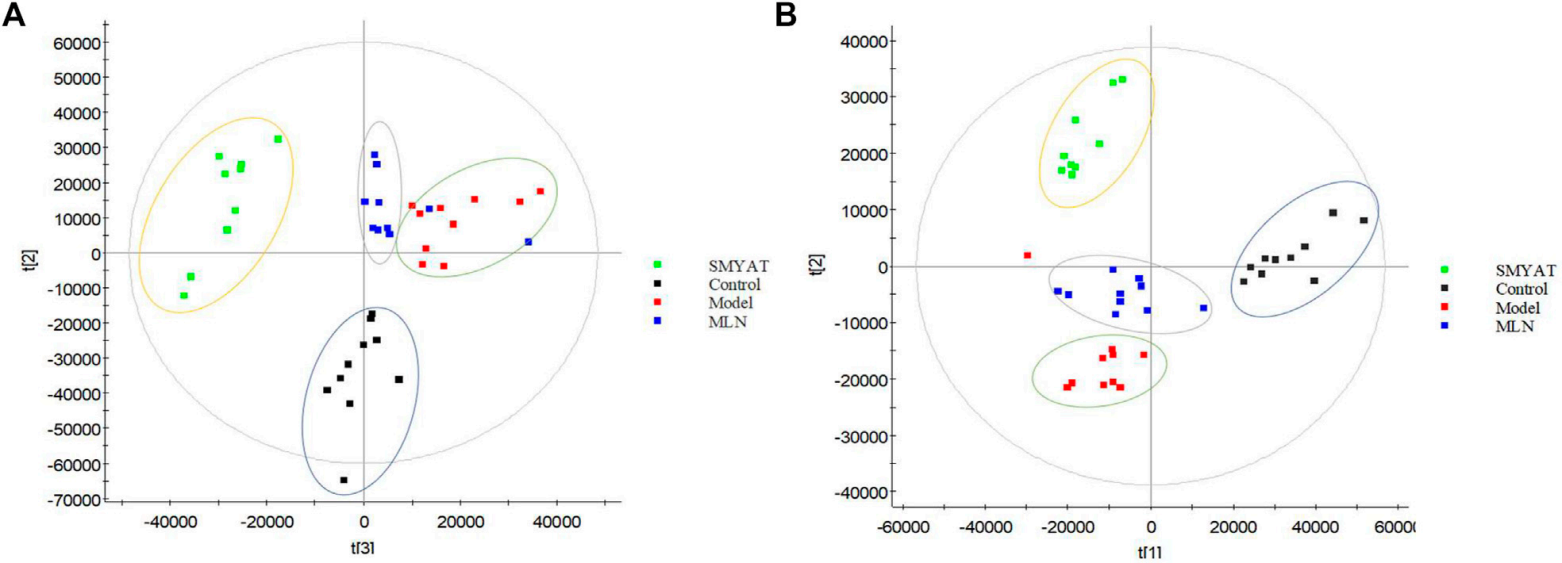
The PCA score plot of control, model, SMYAT, and MLN groups in urine metabolism profile. **(A)** Positive ion mode and **(B)** negative ion mode. Black spot represents the control group, red spot represents the model group, green spot represents the SMYAT group and blue spot represents the MLN group.

The PCA was carried out on the urine data by collected on the 25th day from the four different groups, and the plots obtained (Figure 7), show that there are obvious clusters between each group of data, they are distributed in different positions, especially between the control group and the TAO model group which are far away and can be distinguished significantly. The position of the SMYAT treatment group gradually approached the control groupwhich shows that SMYAT could improve the abnormal metabolic network of TAO model rats.

**FIGURE 8:**
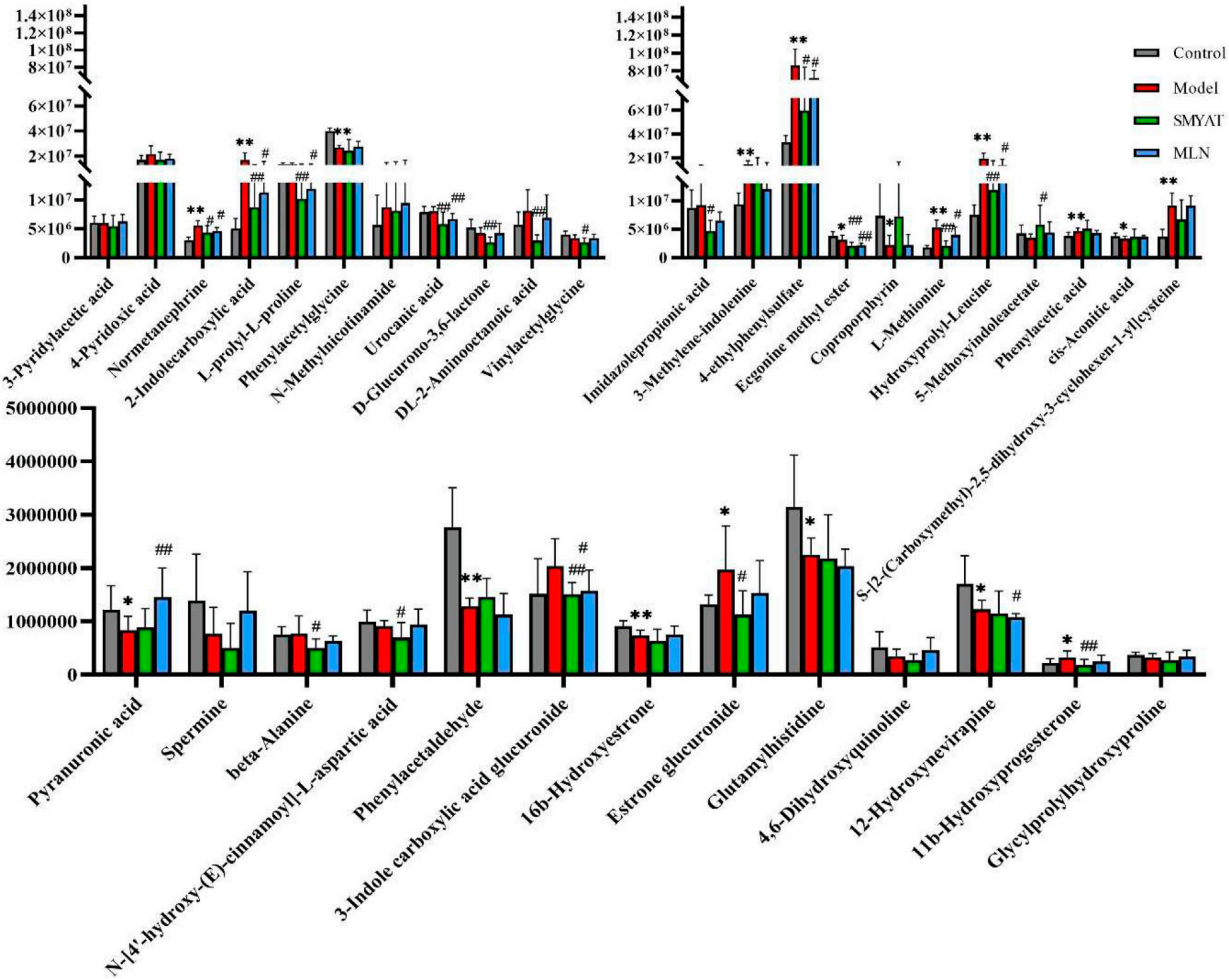
*Significant difference between the control group and model group (*p* < 0.05). **Very significant difference between the control group and model group (*p* < 0.01). ^#^ Significant difference between SMYAT and model groups, *p* < 0.05. ^##^ Very significant difference compared between SMYAT and model groups, *p* < 0.01.

On the 25th day of the experiment, the regulation of biomarkers in rats with thromboangiitis obliterans model by SMYAT was analyzed, among the 35 potential biomarkers that were characterized, SMYAT could be adjusted to 22 biomarkers; respectively including pyranuronic acid, DL-2-aminooctanoic acid, imidazolepropionic acid, L-prolyl-L-prolin, 4-pyridoxic acid, normetanephrine, 2-indolecarboxylic acid, S-[2- (carboxymethyl)-2, 5-dihydroxy-3-cyclohexen-1-yl]cysteine, 3-methylene-indolenine, 4-ethylphenylsulfate, coproporphyrin, hydroxyprolyl-leucine, N-methylnicotinamide, beta-alanine, 3-indole carboxylic acid glucuronide, urocanic acid, phenylacetaldehyde, cis-aconitic acid, estrone glucuronide, 5-methoxyindoleacetate, 11b-hydroxyprogesterone, and L-methionine (Figure 8).

### Metabolic Network Analysis

**FIGURE 9:**
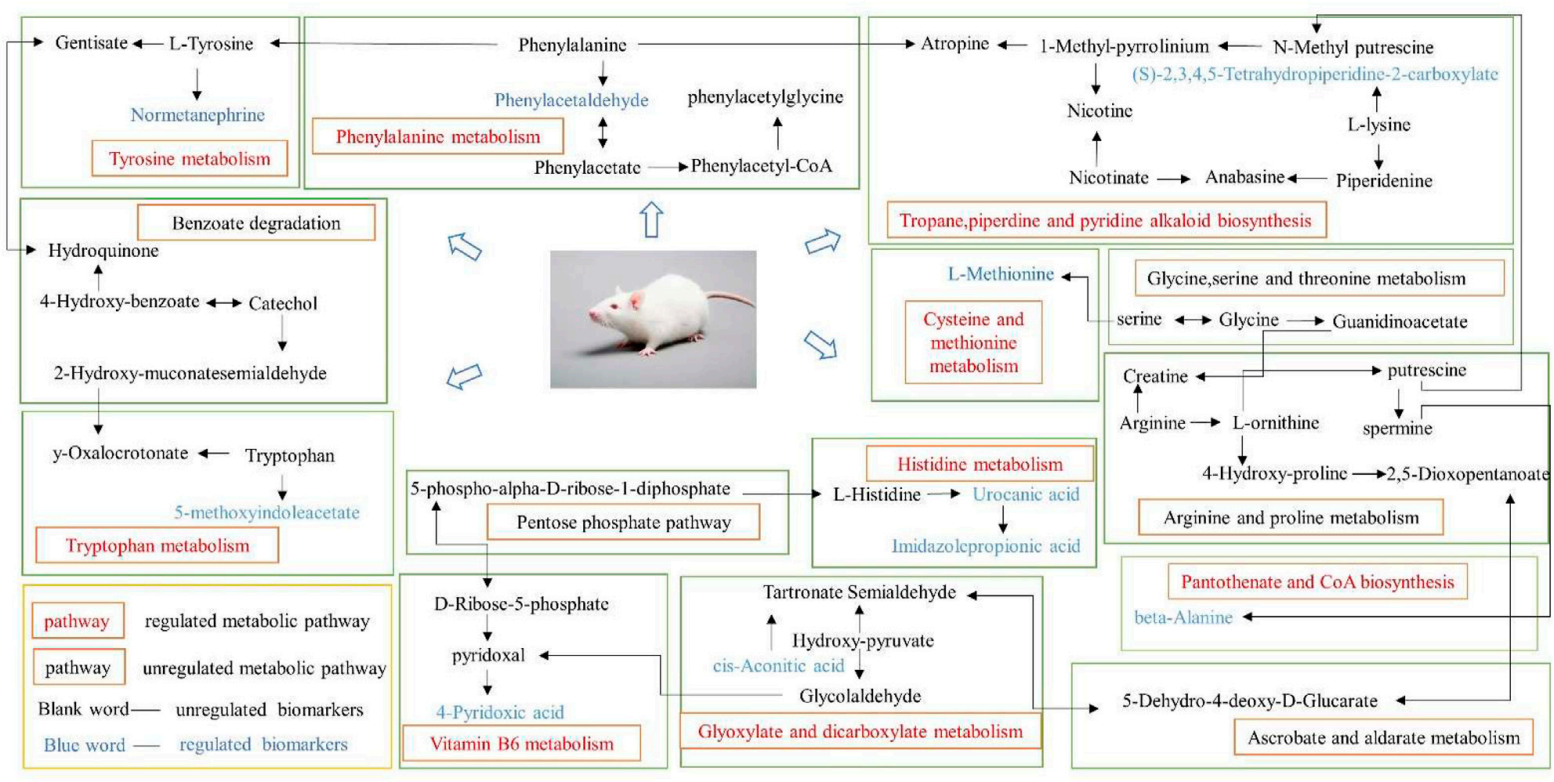
Correlation networks of the potential biomarkers based on the KEGG.

Based on the HMDB, KEGG and the metaboanalyst platform, the metabolic network of SMYAT on thromboangiitis obliterans rats was mapped to clarify its potential mechanism (Figure 9).

## Discussion

Thromboangiitis obliterans is a common vascular disease in clinics, which mainly affects small and medium-sized arteries and veins (Klein-Weigel et al., 2015). Amputation is needed in severe cases. Because its exact pathogenesis and etiology are not clear, there is no radical treatment, causing great pain to patients, but the research on it has not stopped. In this study, metabolomics provides a powerful approach to analyze the urine of TAO model rats and control group rats to study the difference of endogenous small molecule metabolic profiles. On this basis, we found the biomarkers and metabolic pathways regulated by SMYAT, so as to provide ideas for the research on the mechanism of SMYAT in the treatment of thromboangiitis obliterans. In this study, a total of 35 biomarkers of thromboangiitis obliterans model were characterized. In the visualized thermogram of the biomarkers of the thromboangiitis obliterans model, it can be clearly seen that these 35 urine biomarkers can clearly distinguish the control group from the TAO model group, suggesting that the biomarkers we found are significantly related to thromboangiitis obliterans. Among them, 22 biomarkers were adjusted by SMYAT. According to the results of treatment, the affected limbs of rats in the SMYAT treatment group were recovered in varying degrees, the hemorheology and other indicators significantly adjusted back, and the degree of femoral artery lesions improved. This study can show that the metabolomics method can accurately analyze the potential biomarkers and metabolic pathways, so as to achieve a specific, targeted, therapeutic effect. SMYAT can regulate multiple metabolic pathways such as cysteine and methionine metabolism, phenylalanine metabolism, tyrosine metabolism and vitamin B6 metabolism in rats with thromboangiitis obliterans, and achieve good therapeutic effects.

Methionine is an alpha-amino acid, and is found in all organisms ranging from bacteria to plants to animals. It is classified as an aliphatic, non-polar amino acid. In addition to being a substrate for protein synthesis, methionine is an intermediate in transmethylation reactions, serving as the major methyl group donor *in vivo*, including the methyl groups for DNA and RNA intermediates. Acute doses of methionine can lead to acute increases in plasma homocysteine, which can be used as an index of the susceptibility to cardiovascular disease. When present in sufficiently high levels, methionine can act as an atherogen and a metabotoxin. An atherogen is a compound that, when present at chronically high levels, causes atherosclerosis and cardiovascular disease. Methionine oxidation metabolism has a promoting effect on vascular diseases such as atherosclerosis and thrombosis (Gu et al., 2015). Studies have shown that increased homocysteine levels are related to thrombosis. *In vivo*, cysteine is derived from the amino acid containing thiol group formed by methionine metabolism (Undas et al., 2005; Qureshi et al., 2019), and the methionine content is significantly increased in rats with thromboangiitis obliterans, resulting in the metabolic disorder of cysteine and the methionine metabolic pathway, thus promoting thrombosis. After treatment with SMYAT, the content of methionine was significantly reduced. It reminds us that maintaining a stable methionine content plays an important role in preventing thrombosis. The content of normetanephrine in TAO model rats was significantly increased, and normetanephrine was a metabolite produced by catechol-O-methyl transferase. Within humans, normetanephrine participates in a number of enzymatic reactions. It is also involved in the metabolic disorder called transient tyrosinemia. Phenylalanine and tyrosine are used as the precursor of catecholamine and participate in the synthesis of normetanephrine and other substances. In this TAO model, the tyrosine metabolism pathway is disordered, which led to the increase of normetanephrine content. Normetanephrine has a regulatory effect on the degree of vascular tension, which affects the model state (Desjars and Pinaud, 1989). High levels of norepinephrine can reduce blood supply, aggravate ischemia, expand the scope of infarction, and promote thrombosis. After treatment with SMYAT, normetanephrine content is significantly regulated, and the state of blood vessels is improved to prevent thrombosis. The content of hydroxyprolyl-leucine in TAO model rats significantly increased and hydroxyprolyl-leucine was a dipeptide composed of hydroxyproline and leucine. Most of these substances have cell signaling effects and are intermediate products of amino acid degradation pathways. The content of hydroxyprolyl-leucine significantly decreased after treatment with SMYAT, suggesting that the therapeutic effect of SMYAT might be related to the regulation of the hydroxyprolyl-leucine content. 4-ethylphenylsulfate is an organic compound, called phenylsulfate benzene sulfate is a compound containing sulfuric acid group which combines with phenyl. The component in TAO model group is significantly increased and it is significantly regulated after giving SMYAT. Vitamin B6 is a term for a group of interconvertible molecules containing pyridoxine, pyridoxal, pyridoxamine, and their phosphates (Kalle et al., 2016). Vitamin B6 is the main component of a coenzyme in the human body and participates in a variety of metabolic reactions. Vitamin B6 is finally metabolized into 4-pyridoxic through a series of processes in the body (Stanulović et al., 1976). The ratio of 4-pyridoxic acid to pyridoxine is related to cardiovascular disease. It is believed that vitamin B6 in plasma can be metabolized to 4-pyridoxic acid, which increases the risk factors of blood vessels (Obeid et al., 2019). In this research, the vitamin B6 metabolism in TAO model rats was disordered, and the content of 4-pyridoxic acid increased. 4-pyridoxic acid will accumulate in the body, thereby increasing the risk of thrombosis, and it is significantly regulated after giving SMYAT. Many of the biomarkers we have found are related to thrombosis and have been significantly adjusted. This also shows that SMYAT has a significant effect in the treatment of this disease and has the value of in-depth analysis.

Metabolomics technology is used to characterize the metabolic profile of syndromes and find biomarkers from the level of endogenous small molecule metabolism (Liang et al., 2015c; Qiu et al., 2017, 2020). Because of its high sensitivity, it is very effective in the identification of biomarkers, so it also reflects a new strategy for the diagnosis of thromboangiitis obliterans. This study is the first to apply the technology of urine metabolomics to the study of the mechanism of thromboangiitis obliterans, which provides an innovative idea for the study of the mechanism of thromboangiitis obliterans. SMYAT has a good regulatory effect on thromboangiitis obliterans. Based on UPLC-MS/MS technology, the metabolomics method was used to evaluate the overall therapeutic effect of SMYAT and study its potential molecular mechanism. This experiment provides strong evidence for the mechanism of SMYAT in treating thromboangiitis obliterans and a reliable method for exploring the mechanism of traditional Chinese medicine.

## Conclusion

In our research, the rat model of thromboangiitis obliterans was established by injection of sodium laurate, and the effect of SMYAT was evaluated by combining the evaluation indexes of hemorheology, histopathology, and metabolomic methods. In the rat model of thromboangiitis obliterans, there are 35 endogenous urinary metabolites as potential biomarkers. The metabolic pathways include vitamin B6 metabolism, phenylalanine metabolism, cysteine and methionine metabolism and tyrosine metabolism, and so forth. These findings add to our understanding of the pathogenesis of thromboangiitis obliterans and reveal the molecular basis of the efficacy of SMYAT in the treatment of thromboangiitis obliterans through metabolomic techniques. SMYAT can improve thromboangiitis obliterans and is a promising candidate drug for thromboangiitis obliterans.

## Data Availability

The original contributions presented in the study are included in the article/Supplementary Material, further inquiries can be directed to the corresponding authors.
