# Supplementary material for: Therapeutic Effect and Mechanism of Si-Miao-Yong-An-Tang on Thromboangiitis Obliterans Based on the Urine Metabolomics Approach
Source: Front Pharmacol. 2022 Feb 22;13:827733. doi: 10.3389/fphar.2022.827733 (PMC8902467; doi:10.3389/fphar.2022.827733)
Supplement: Supplementary file 1 [file Table1.DOCX]

**TABLE S1** Identification of urinary biomarkers in TAO model groups.

| **No.** | **Retention Time** | **Mass** | **[M-H]^-^/[M+H]^+^** | **Formular** | **MS/MS Fragments** | **Identified** |
| --- | --- | --- | --- | --- | --- | --- |
| 1 | 0.56 | 203.2233 | [M+H]^+^ | C_10_H_26_N_4_ | 145,129,110,53 | Spermine |
| 2 | 0.63 | 193.034 | [M-H]^-^ | C_6_H_10_O_7_ | 129,111,75 | Pyranuronic acid |
| 3 | 0.69 | 137.0716 | [M+H]^+^ | C_7_H_8_N_2_O | 94,78,65 | N-Methylnicotinamide |
| 4 | 0.76 | 90.0555 | [M+H]^+^ | C_3_H_7_NO_2_ | 70,55 | beta-Alanine |
| 5 | 0.94 | 173.0084 | [M-H]^-^ | C_6_H_6_O_6_ | 131,117,96 | cis-Aconitic acid |
| 6 | 1.01 | 175.0240 | [M-H]^-^ | C_6_H_8_O_6_ | 115,87,55 | D-Glucurono-3,6-lactone |
| 7 | 1.12 | 160.1336 | [M+H]^+^ | C_8_H_17_NO_2_ | 144,126,114,96,59 | DL-2-Aminooctanoic acid |
| 8 | 1.47 | 245.1500 | [M+H]^+^ | C_11_H_20_N_2_O_4_ | 209,185,163,148,125,96,84 | Hydroxyprolyl-Leucine |
| 9 | 1.48 | 139.0509 | [M+H]^+^ | C_6_H_6_N_2_O_2_ | 107,77,53 | Urocanic acid |
| 10 | 1.82 | 143.0586 | [M+H]^+^,[M+Na]^+^ | C_6_H_9_NO_3_ | 128,101,84,71,54 | Vinylacetylglycine |
| 11 | 2.18 | 141.0664 | [M+H]^+^ | C_6_H_8_N_2_O_2_ | 123,95,81 | Imidazolepropionic acid |
| 12 | 2.78 | 212.1163 | [M+H]^+^,[M+Na]^+^ | C_10_H_16_N_2_O_3_ | 194,169,140,116,70 | L-prolyl-L-proline |
| 13 | 2.88 | 190.0490 | [M+FA-H]^-^ | C_5_H_11_NO_2_S | 135,118,73 | L-Methionine |
| 14 | 2.94 | 324.0731 | [M+FA-H]^-^ | C_13_H_13_NO_6_ | 134,124,85 | N-[4'-hydroxy- (E)-cinnamoyl]-L-aspartic acid |
| 15 | 2.95 | 137.0479 | [M-H]^-^,[M+FA-H]^-^ | C_7_H_8_NO_2_ | 120,108,93 | 3-Pyridylacetic acid |
| 16 | 2.96 | 184.0611 | [M+H]^+^ | C_8_H_9_NO_4_ | 166,148 | 4-Pyridoxic acid |
| 17 | 3.24 | 330.1296 | [M+FA-H]^-^ | C_12_H_19_N_3_O_5_ | 226,175,113,85 | Glycylprolylhydroxyproline |
| 18 | 3.53 | 184.0972 | [M+H]^+^ | C_9_H_13_NO_3_ | 170,137,124 | Normetanephrine |
| 19 | 3.58 | 137.0601 | [M+H]^+^ | C_8_H_8_O_2_ | 122,93,77 | Phenylacetic acid |
| 20 | 3.89 | 162.0556 | [M+H]^+^ | C_9_H_7_NO_2_ | 144,116,89 | 2-Indolecarboxylic acid |
| 21 | 4.50 | 336.0755 | [M+FA-H]^-^ | C_11_H_17_NO_6_S | 175,160,129,113,85 | S-[2- (Carboxymethyl)-2,5-dihydroxy-3-cyclohexen-1-yl]cysteine |
| 22 | 5.14 | 193.0746 | [M+H]^+^,[M+Na]^+^ | C_10_H_11_NO_3_ | 150,133,117,91 | Phenylacetylglycine |
| 23 | 5.16 | 336.0719 | [M-H]^-^ | C_15_H_15_NO_8_ | 193,175,113,85 | 3-Indole carboxylic acid glucuronide |
| 24 | 5.39 | 121.0652 | [M+H]^+^ | C_8_H_8_O | 106,92,77 | Phenylacetaldehyde |
| 25 | 5.44 | 285.1484 | [M-H]^-^ | C_18_H_22_O_3_ | 191,178,147,117,97 | 16b-Hydroxyestrone |
| 26 | 5.53 | 445.1879 | [M-H]^-^ | C_24_H_30_O_8_ | 304,279,92 | Estrone glucuronide |
| 27 | 5.77 | 285.1184 | [M+H]^+^ | C_11_H_16_N_4_O_5_ | 243,225,207,189,161,147,133 | Glutamylhistidine |
| 28 | 6.15 | 160.0396 | [M-H]^-^ | C_9_H_7_NO_2_ | 138,118,95 | 4,6-Dihydroxyquinoline |
| 29 | 6.33 | 204.0658 | [M-H]^-^ | C_11_H_11_NO_3_ | 191,173,145,97 | 5-Methoxyindoleacetate |
| 30 | 6.56 | 281.1053 | [M-H]^-^ | C_15_H_14_N_4_O_2_ | 147,120,95 | 12-Hydroxynevirapine |
| 31 | 6.64 | 130.0656 | [M+H]^+^ | C_9_H_7_N | 115,103,91,77 | 3-Methylene-indolenine |
| 32 | 6.72 | 201.0217 | [M-H]^-^ | C_8_H_10_O_4_S | 121,106,79 | 4-ethylphenylsulfate |
| 33 | 6.92 | 200.1282 | [M+H]^+^ | C_10_H_17_NO_3_ | 183,159,132,91 | Ecgonine methyl ester |
| 34 | 7.85 | 331.1899 | [M-H]^-^ | C_20_H_28_O_4_ | 207,191,178, | 11b-Hydroxyprogesterone |
| 35 | 8.05 | 655.2761 | [M+H]^+^ | C_36_H_38_N_4_O_8_ | 596,537,523,478,405 | Coproporphyrin |
