# Supplementary material for: Therapeutic Effect and Mechanism of Si-Miao-Yong-An-Tang on Thromboangiitis Obliterans Based on the Urine Metabolomics Approach
Source: Front Pharmacol. 2022 Feb 22;13:827733. doi: 10.3389/fphar.2022.827733 (PMC8902467; doi:10.3389/fphar.2022.827733)
Supplement: Supplementary file 2 [file DataSheet3.docx]

**Supplementary materials 3**


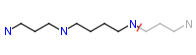

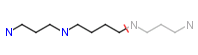

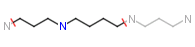


1--Spermine (C_10_H_26_N_4_)


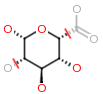

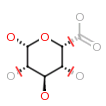

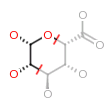


2--Pyranuronic acid (C_6_H_10_O_7_)


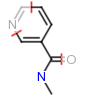

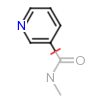

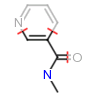


3--N-Methylnicotinamide (C_7_H_8_N_2_O)


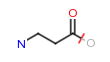

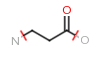


4-- beta-Alanine (C_3_H_7_NO_2_)


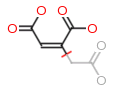

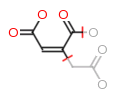

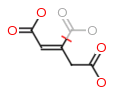


5--cis-Aconitic acid (C_6_H_6_O_6_)


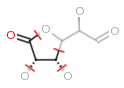

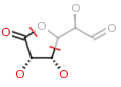

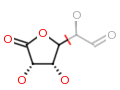


6--D-Glucurono-3,6-lactone (C_6_H_8_O_6_)


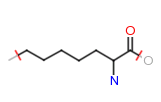

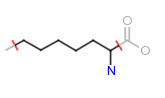

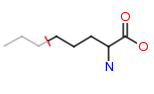

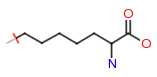


7--DL-2-Aminooctanoic acid (C_8_H_17_NO_2_)


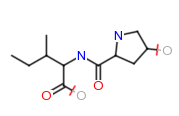

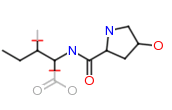

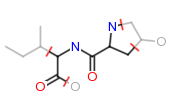

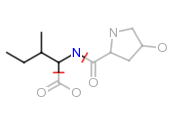

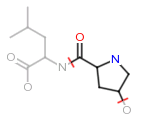


8--Hydroxyprolyl-Leucine (C_11_H_20_N_2_O_4_)


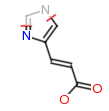

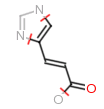


9--Urocanic acid (C_6_H_6_N_2_O_2_)


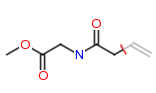

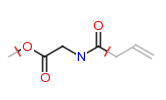

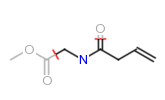

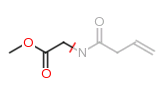


10--Vinylacetylglycine (C_6_H_9_NO_3_)


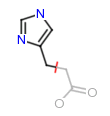

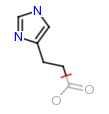

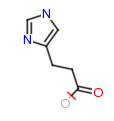


11--Imidazolepropionic acid (C_6_H_8_N_2_O_2_)


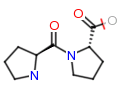

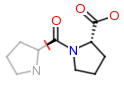

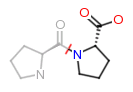

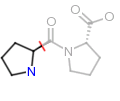


12--L-prolyl-L-proline (C_10_H_16_N_2_O_3_)


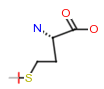

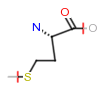

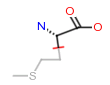


13--L-Methionine (C_5_H_11_NO_2_S)


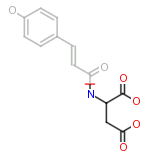

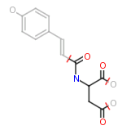

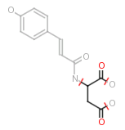


14--N-[4'-hydroxy- (E)-cinnamoyl]-L-aspartic acid (C_13_H_13_NO_6_)


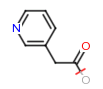

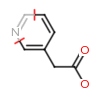

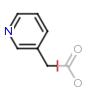


15--3-Pyridylacetic acid (C_7_H_8_NO_2_)


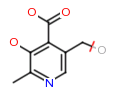

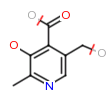


16--4-Pyridoxic acid (C_8_H_9_NO_4_)


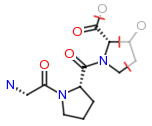

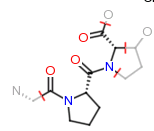

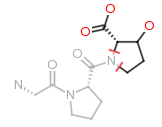

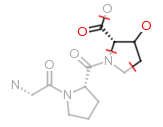


17--Glycylprolylhydroxyproline (C_12_H_19_N_3_O_5_)


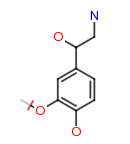

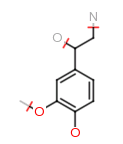

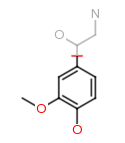


18--Normetanephrine (C_9_H_13_NO_3_)


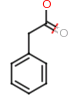

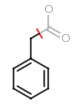

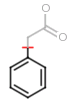


19--Phenylacetic acid (C_8_H_8_O_2_)


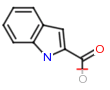

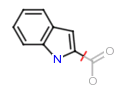

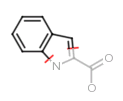


20--2-Indolecarboxylic acid (C_9_H_7_NO_2_)


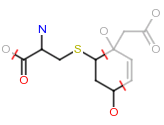

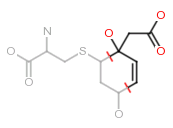

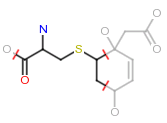

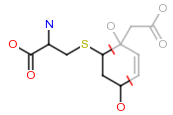


21--S-[2- (Carboxymethyl)-2,5-dihydroxy-3-cyclohexen-1-yl]cysteine (C_11_H_17_NO_6_S)


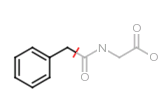

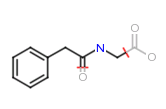

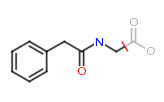

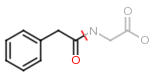


22--Phenylacetylglycine (C_10_H_11_NO_3_)


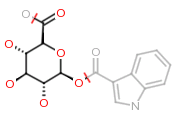

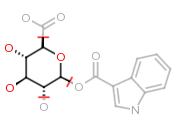

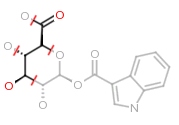

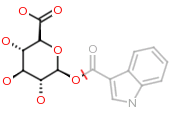


23--3-Indole carboxylic acid glucuronide (C_15_H_15_NO_8_)


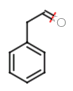

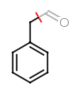


24--Phenylacetaldehyde (C_8_H_8_O)


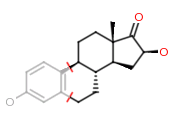

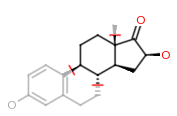

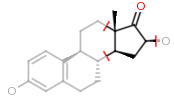


25--16b-Hydroxyestrone (C_18_H_22_O_3_)


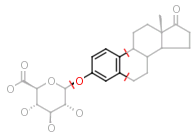

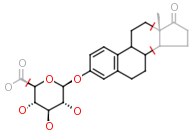

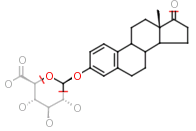


26--Estrone glucuronide (C_24_H_30_O_8_)


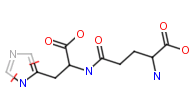

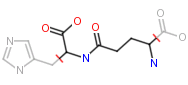

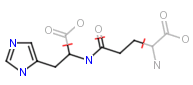

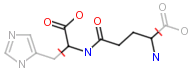


27--Glutamylhistidine (C_11_H_16_N_4_O_5_)


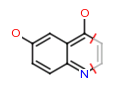

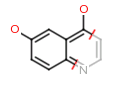

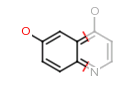


28--4,6-Dihydroxyquinoline (C_9_H_7_NO_2_)


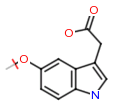

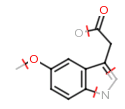

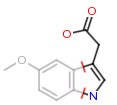


29--5-Methoxyindoleacetate (C_11_H_11_NO_3_)


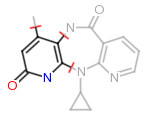

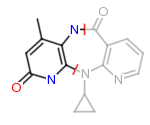

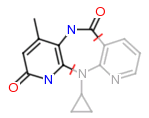


30--12-Hydroxynevirapine (C_15_H_14_N_4_O_2_)


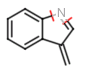

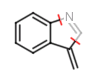

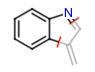

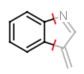


31--3-Methylene-indolenine (C_9_H_7_N)

32--4-ethylphenylsulfate (C_8_H_10_O_4_S)

33--Ecgonine methyl ester (C_10_H_17_NO_3_)

34--11b-Hydroxyprogesterone (C_20_H_28_O_4_)

35--Coproporphyrin (C_36_H_38_N_4_O_8_)
