# Supplementary material for: Therapeutic Effect and Mechanism of Si-Miao-Yong-An-Tang on Thromboangiitis Obliterans Based on the Urine Metabolomics Approach
Source: Front Pharmacol. 2022 Feb 22;13:827733. doi: 10.3389/fphar.2022.827733 (PMC8902467; doi:10.3389/fphar.2022.827733)
Supplement: Supplementary file 3 [file DataSheet2.docx]

**Supplementary materials 2**

We have carried out quality control on the samples of Si-Miao-Yong-An-Tang. The following contents are taken as an example to show the chromatogram and detection conditions of the main components of Si-Miao-Yong-An-Tang. The chromatograms of Si-Miao-Yong-An-Tang samples and Chemical reference standards are shown in **Figure 1**. Six samples of Si-Miao-Yong-An-Tang were prepared in parallel to obtain chlorogenic acid, ferulic acid, isochlorogenic acid A, isochlorogenic acid C and other chemical components. The results of the content are shown in **Table 1**-**4**. The results show that the RSD values of each component we determined are all less than 3.0%, indicating that the quality of Si-Miao-Yong-An-Tang samples is stable.

HPLC conditions：
Chromatographic column: DIKMA Diamonsil C18（200×4.6mm，5μm）

Column temperature: 30℃

Injection volume: 20μL

Detection wavelength: 327,316nm

Flow rate: 1mL/min

Mobile phase: acetonitrile (A), water with 0.05% phosphoric acid (B).

The gradient was as follows: 0~5min, 11%A; 5~8min,11-21%A; 8~19min, 21-26%A;

Sample of Si-Miao-Yong-An-Tang

Chemical reference standards

Chlorogenic acid

Ferulic acid

Isochlorogenic acid A

Isochlorogenic acid C

**Figure 1** Sample quality control chromatogram of Si-Miao-Yong-An-Tang

**Table 1** Chlorogenic acid content table

| Serial number | content (%) | Average content (%) | RSD (%) |
| --- | --- | --- | --- |
| 1 | 1.36 | 1.37 | 1.11 |
| 2 | 1.37 |  |  |
| 3 | 1.38 |  |  |
| 4 | 1.38 |  |  |
| 5 | 1.40 |  |  |
| 6 | 1.36 |  |  |

**Table 2** Ferulic acid content table

| Serial number | content (%) | Average content (%) | RSD (%) |
| --- | --- | --- | --- |
| 1 | 1.00 | 0.98 | 1.49 |
| 2 | 0.96 |  |  |
| 3 | 0.97 |  |  |
| 4 | 0.99 |  |  |
| 5 | 0.96 |  |  |
| 6 | 0.99 |  |  |

**Table 3** Isochlorogenic acid A content table

| Serial number | content (%) | Average content (%) | RSD (%) |
| --- | --- | --- | --- |
| 1 | 0.23 | 0.24 | 2.99 |
| 2 | 0.23 |  |  |
| 3 | 0.25 |  |  |
| 4 | 0.23 |  |  |
| 5 | 0.25 |  |  |
| 6 | 0.24 |  |  |

**Table 4** Isochlorogenic acid C content table

| Serial number | content (%) | Average content (%) | RSD (%) |
| --- | --- | --- | --- |
| 1 | 0.30 | 0.31 | 2.28 |
| 2 | 0.31 |  |  |
| 3 | 0.32 |  |  |
| 4 | 0.31 |  |  |
| 5 | 0.32 |  |  |
| 6 | 0.31 |  |  |
