# Supplementary material for: Therapeutic Effect and Mechanism of Si-Miao-Yong-An-Tang on Thromboangiitis Obliterans Based on the Urine Metabolomics Approach
Source: Front Pharmacol. 2022 Feb 22;13:827733. doi: 10.3389/fphar.2022.827733 (PMC8902467; doi:10.3389/fphar.2022.827733)
Supplement: Supplementary file 4 [file DataSheet1.docx]

**Supplementary materials 1**


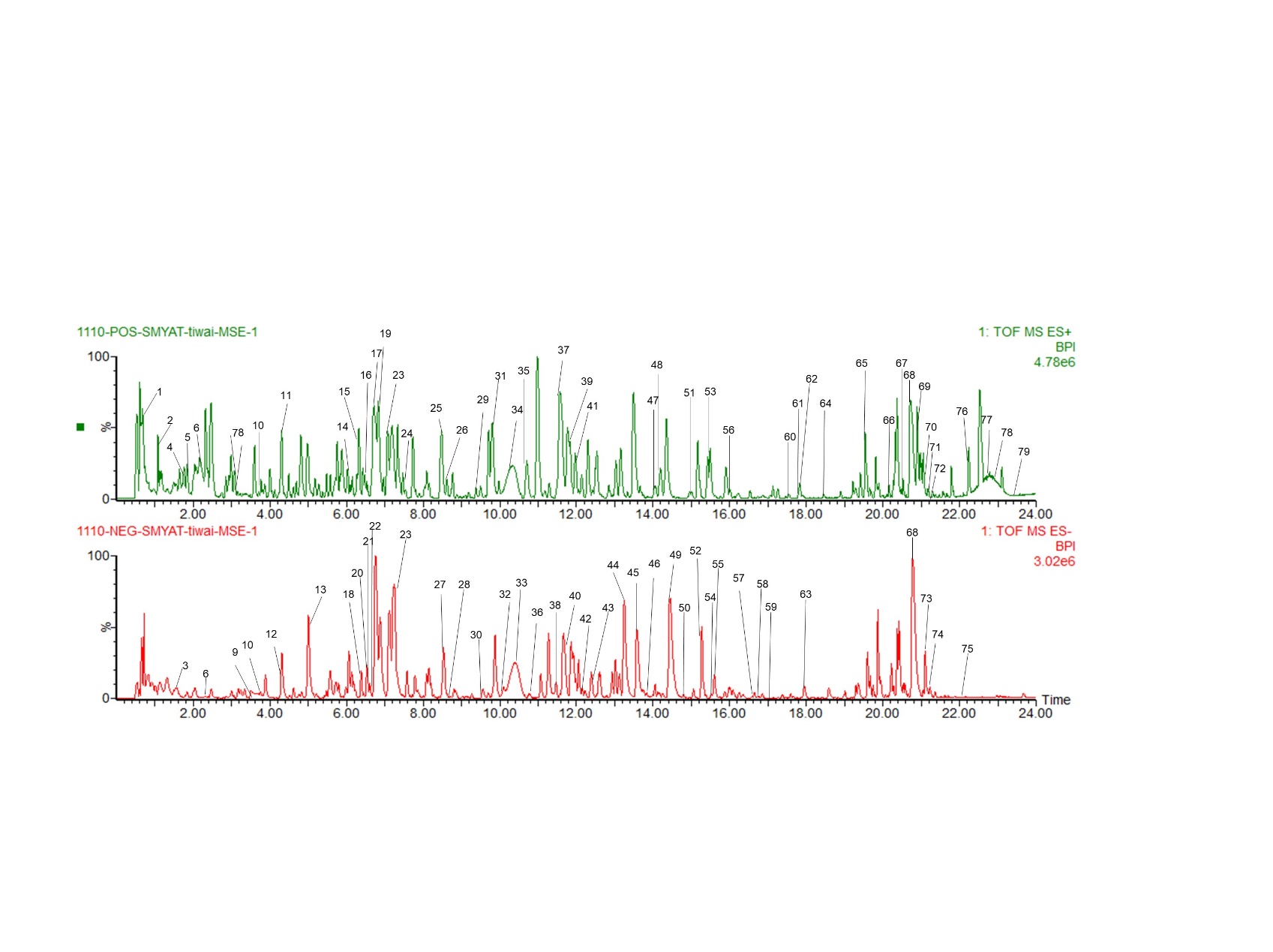


**Figure 1** Base peak ion (BPI) chromatograms of Si-Miao-Yong-An-Tang samples under different ion modes

**Table 1** Specific information of Si-Miao-Yong-An-Tang compounds

| No． | Observed RT (min) | Component name | Observed neutral mass (Da) | Adducts | Iformula | Neutral mass (Da) | Observed m/z | Mass error (mDa) | Mass error (ppm) | Detector counts | Fragments | Original |
| --- | --- | --- | --- | --- | --- | --- | --- | --- | --- | --- | --- | --- |
| 1 | 0.68 | Asparagine | 132.0542 | +H | C_4_H_8_N_2_O_3_ | 132.05349 | 133.0615 | 0.7 | 5.3 | 5901 | 87.04[C_4_H_7_O_2_]，102.05[C_4_H_8_NO_2_]； | A |
| 2 | 1.16 | Nicotinic acid | 123.0326 | +H | C_6_H_5_NO_2_ | 123.03203 | 124.0399 | 0.6 | 4.6 | 59573 | 80.04[C_5_H_6_N]； | C |
| 3 | 1.68 | Succinic acid | 118.02661 | -H | C_4_H_6_O_4_ | 118.0269 | 117.0196 | 0.3 | 2.3 | 1636 | 73.03[C_3_H_5_O_2_]； | A |
| 4 | 1.81 | Uracil | 112.0275 | +H | C_4_H_4_N_2_O_2_ | 112.02728 | 113.0348 | 0.3 | 2.3 | 18545 | 112.02[C_4_H_4_N_2_O_2_]； | C |
| 5 | 1.83 | Licochalcone A | 338.1499 | +H | C_21_H_22_O_4_ | 338.15181 | 339.1572 | -1.9 | -5.6 | 3008 | 161.06[C_10_H_9_O_2_]，268.07[C_16_H_12_O_4_]； | D |
| 6 | 2.32 | Adenine | 135.0551 | +H | C_5_H_5_N_5_ | 135.0545 | 136.0623 | 0.6 | 4.2 | 816418 | 119.03[C_5_H_3_N_4_]，94.03[C_4_H_4_N_3_]； | C |
| 6 | 2.33 | Adenine | 135.0545 | -H | C_5_H_5_N_5_ | 135.0544 | 134.0471 | -0.1 | -0.7 | 7059 | 107.03[C_4_H_3_N_4_]，119.03[C_5_H_3_N_4_]； | C |
| 7 | 3.04 | Catalpol | 362.1192 | +H | C_15_H_22_O_10_ | 362.1213 | 363.1265 | -2.1 | -5.7 | 47395 | 120.08[C_5_H_12_O_3_]，146.06[C_6_H_10_O_4_]； | B |
| 8 | 3.16 | 3-Feruloylquinic acid | 368.109 | +H | C_17_H_20_O_19_ | 368.11073 | 369.1163 | -1.7 | -4.6 | 117636 | 149.06[C_9_H_9_O_2_]； | B |
| 9 | 3.67 | Ningpogoside A | 332.14712 | -H | C_15_H_24_O_8_ | 332.1486 | 331.1414 | 1.5 | 4.6 | 1710 | 229.07[C_10_H_13_O_6_]，179.05[C_6_H_11_O_6_]； | A |
| 10 | 3.87 | Secologanoside | 390.1168 | +H | C_16_H_22_O_11_ | 390.11621 | 391.1241 | 0.6 | 1.6 | 52739 | 151.03[C_8_H_7_O_3_]，211.06[C_10_H_11_O_5_]； | B |
| 10 | 3.88 | Secologanoside | 390.11621 | -H | C_16_H_22_O_11_ | 390.1169 | 389.1096 | 0.7 | 1.8 | 237548 | 121.06[C_8_H_9_O]，165.05[C_9_H_9_O_3_]； | B |
| 11 | 4.29 | 4-Ethyl-m-dihydroxybenzene | 138.0687 | +H | C_8_H_10_O | 138.06808 | 139.076 | 0.6 | 4.5 | 16644 | 109.02[C_6_H_5_O_2_]，121.06[C_8_H_9_O]； | C |
| 12 | 4.32 | Harpagide | 364.13695 | -H | C_15_H_24_O_10_ | 364.1375 | 363.1302 | 0.6 | 1.5 | 490398 | 183.06[C_9_H_11_O_4_]，201.07[C_9_H_13_O_5_]； | A |
| 13 | 5.01 | Chlorogenic acid | 354.09508 | -H | C_16_H_18_O_9_ | 354.0957 | 353.0884 | 0.6 | 1.6 | 715963 | 191.05[C_7_H_10_O_6_]，179.03[C_9_H_7_O_4_]； | B |
| 14 | 6.01 | 6-O-α-D-Galactopyra-nosylharpagoside | 656.2262 | +H | C_13_H_24_O_11_ | 656.23164 | 657.2335 | -5.4 | -8.2 | 5790 | 166.08[C_6_H_14_O_5_]，221.06[C_8_H_13_O_7_]； | A |
| 15 | 6.31 | Phthalic anhydride | 148.0163 | +H | C_8_H_4_O_3_ | 148.01604 | 149.0236 | 0.2 | 1.6 | 14689 | 107.04[C_7_H_7_O]，135.04[C_8_H_7_O_2_]； | C |
| 16 | 6.44 | Buergeriside C1 | 324.1217 | +H | C_16_H_20_O_7_ | 324.1209 | 325.129 | 0.8 | 2.4 | 1744 | 324.12[C_16_H_20_O_7_]; | A |
| 17 | 6.5 | Anisic acid | 152.0478 | +H | C_8_H_8_O_3_ | 152.04734 | 153.0551 | 0.5 | 3 | 47894 | 109.06[C_7_H_9_O]，137.05[C_8_H_9_O_2_]； | C |
| 18 | 6.54 | Ningpogenin | 170.09429 | -H | C_9_H_14_O_3_ | 170.0945 | 169.0872 | 0.2 | 1 | 765 | 121.06[C_8_H_9_O]，151.07[C_9_H_11_O_2_]； | A |
| 19 | 6.65 | Ningposide D | 366.1292 | +H | C_18_H_22_O_8_ | 366.13147 | 367.1365 | -2.2 | -6.1 | 4077 | 151.03[C_8_H_7_O_3_]，177.05[C_10_H_9_O_3_]； | A |
| 20 | 6.7 | Guaiacol | 124.05243 | -H | C_7_H_8_O_2_ | 124.0524 | 123.0451 | 0 | -0.3 | 3074 | 123.05[C_7_H_8_O_2_]； | C |
| 21 | 6.72 | 3-Methoxy-4-hydroxybenzoic acid | 168.04226 | -H | C_8_H_8_O_4_ | 168.0421 | 167.0348 | -0.2 | -1 | 16478 | 123.04[C_7_H_7_O_2_]； | A |
| 22 | 6.77 | 3,4-Dihydroxycinnamic acid | 180.04226 | -H | C_9_H_8_O_4_ | 180.0426 | 179.0354 | 0.4 | 2.2 | 3301 | 161.02[C_9_H_5_O_3_]，133.02[C_8_H_5_O_2_]； | B |
| 23 | 7.19 | Secologanic acid | 374.1219 | +H | C_16_H_22_O_10_ | 374.1213 | 375.1292 | 0.6 | 1.7 | 249715 | 151.03[C_8_H_7_O_3_]，195.06[C_10_H_11_O_4_]； | B |
| 23 | 7.23 | Secologanic acid | 374.1213 | -H | C_16_H_22_O_10_ | 374.1225 | 373.1152 | 1.2 | 3.1 | 1413060 | 193.05[C_10_H_9_O_4_]，149.06[C_9_H_9_O_2_]； | B |
| 24 | 7.52 | Vanillin | 152.0476 | +H | C_8_H_8_O_3_ | 152.04734 | 153.0549 | 0.3 | 1.8 | 22599 | 107.04[C_7_H_7_O]，135.04[C_8_H_7_O_2_]； | C |
| 25 | 8.46 | Licoflavone A | 322.1173 | +H | C_20_H_18_O_4_ | 322.12051 | 323.1246 | -3.2 | -9.8 | 2882 | 95.04[C_6_H_7_O]，133.06[C_9_H_9_O]； | D |
| 26 | 8.48 | p-Methoxycinnamic acid | 178.0636 | +H | C_10_H_10_O_3_ | 178.06299 | 179.0708 | 0.6 | 3.2 | 286129 | 95.04[C_6_H_7_O]，133.06[C_9_H_9_O]； | A |
| 27 | 8.53 | Luteolin-7-O-α-D-glucoside | 450.11621 | -H | C_21_H_22_O_11_ | 450.118 | 449.1108 | 1.8 | 4 | 3654 | 337.09[C_16_H_17_O8]，125.02[C_6_H_5_O_3_]； | B |
| 28 | 8.54 | Secoxyloganin | 404.13186 | -H | C_17_H_24_O_11_ | 404.1321 | 403.1249 | 0.3 | 0.7 | 547248 | 357.12[C_16_H_21_O_9_]，225.07[C_11_H_13_O_5_]； | B |
| 29 | 9.46 | Verbascoside | 624.2109 | +H | C_29_H_36_O_15_ | 624.20542 | 625.2182 | 5.5 | 8.8 | 47425 | 492.17[C_24_H_28_O_11_]，418.14[C_18_H_26_O_11_]； | A |
| 30 | 9.56 | 4-Feruloylquinic acid | 368.11073 | -H | C_17_H_20_O_9_ | 368.1114 | 367.1041 | 0.7 | 1.9 | 107545 | 191.05[C_7_H_11_O_6_]，173.04[C_7_H_9_O_5_]； | B |
| 31 | 9.96 | Corymbosin | 358.1028 | +H | [C_19_H_18_O_7_](https://pubchem.ncbi.nlm.nih.gov/#query=C19H18O7) | 358.10525 | 359.1101 | -2.4 | -6.8 | 2399 | 167.07[C_9_H_11_O_3_]； | B |
| 32 | 10.07 | Decaffeoylacteoside | 462.17373 | -H | C_20_H_30_O_12_ | 462.1749 | 461.1676 | 1.2 | 2.6 | 26555 | 379.13[C_19_H_23_O_8_]，191.05[C_7_H_11_O_6_]； | A |
| 33 | 10.1 | 7-epi-Vogeloside | 388.13695 | -H | C_17_H_24_O_10_ | 388.1383 | 387.131 | 1.4 | 3.6 | 5377 | 155.03[C_7_H_7_O_4_]，225.08[C_11_H_13_O_5_]； | B |
| 34 | 10.33 | Ningposide A | 382.1252 | +H | C_18_H_22_O_9_ | 382.12638 | 383.1325 | -1.2 | -3 | 2044 | 107.04[C_7_H_7_O]，165.05[C_9_H_9_O_3_]； | A |
| 35 | 10.71 | 7-Methoxycoumarin | 176.048 | +H | C_10_H_8_O_3_ | 176.04734 | 177.0552 | 0.6 | 3.5 | 517216 | 145.02[C_9_H_5_O_2_]，163.03[C_9_H_7_O_3_]； | D |
| 36 | 10.78 | Ferulic acid | 194.05791 | -H | C_10_H_10_O_4_ | 194.0576 | 193.0503 | -0.3 | -1.5 | 45583 | 134.03[C_8_H_6_O_2_]，178.02[C_9_H_6_O_4_]； | D |
| 37 | 11.58 | Liquiritigenin | 256.0742 | +H | C_15_H_12_O_4_ | 256.07356 | 257.0815 | 0.7 | 2.7 | 2865606 | 137.02[C_7_H_5_O_3_]，163.04[C_9_H_7_O_3_]； | D |
| 38 | 11.67 | Neoliquiritin | 418.12638 | -H | C_21_H_22_O_9_ | 418.1267 | 417.1194 | 0.3 | 0.8 | 1091186 | 135.00[C_7_H_3_O_3_]，255.06[C_15_H_11_O_4_]； | D |
| 39 | 11.79 | Isoliquiritigenin | 256.0744 | +H | C_15_H_12_O_4_ | 256.07356 | 257.0817 | 0.8 | 3.2 | 1854043 | 137.02[C_7_H_5_O_3_]，151.04[C_8_H_7_O_3_]； | D |
| 40 | 11.87 | Licurazide | 550.16864 | -H | C_26_H_30_O_13_ | 550.1688 | 549.1616 | 0.2 | 0.3 | 996896 | 255.06[C_15_H_11_O_4_]，473.10[C_23_H_21_O_11_]； | D |
| 41 | 11.99 | Quercetin-3-O-β-D-glucopyranoside | 464.0961 | +H | C_21_H_20_O_12_ | 464.09548 | 465.1034 | 0.7 | 1.4 | 302070 | 153.01[C_7_H_5_O_4_]，303.05[C_15_H_11_O_7_]； | B |
| 42 | 12.16 | Sibirioside A | 472.15808 | -H | C_21_H_28_O_12_ | 472.1592 | 471.152 | 1.2 | 2.5 | 119692 | 147.04[C_9_H_7_O_2_]，323.09[C_12_H_19_O_10_]； | A |
| 43 | 12.47 | Sweroside | 372.14203 | -H | C_17_H_24_O_9_ | 372.1425 | 371.1353 | 0.5 | 1.3 | 1250 | 193.05[C_10_H_9_O_4_]，173.04[C_7_H_9_O_5_]； | B |
| 44 | 13.25 | Umbelliferone | 162.03169 | -H | C_9_H_6_O_3_ | 162.0317 | 161.0244 | 0 | 0 | 4650 | 137.02[C_7_H_5_O_3_]，93.03[C_6_H_5_O]； | D |
| 45 | 13.76 | Luteolin-7-O-β-D-galactoside | 448.10056 | -H | C_21_H_20_O_11_ | 448.1017 | 447.0944 | 1.2 | 2.6 | 51266 | 284.03[C_15_H_8_O_6_]，151.00[C_7_H_3_O_4_]； | B |
| 46 | 13.85 | Ningposide C | 352.11582 | -H | C_17_H_20_O_8_ | 352.1169 | 351.1096 | 1.1 | 3.1 | 1525 | 191.05[C_7_H_11_O_6_]，179.03[C_9_H_7_O_4_]； | A |
| 47 | 14.01 | Liquiritin | 418.1289 | +H | C_21_H_22_O_9_ | 418.12638 | 419.1362 | 2.5 | 6 | 6459 | 139.03[C_7_H_7_O_3_]，163.03[C_9_H_7_O_3_]； | D |
| 48 | 14.19 | Isoeugenol | 164.0842 | +H | C_11_H_14_O_2_ | 164.08373 | 165.0914 | 0.4 | 2.6 | 10513 | 95.04[C_6_H_7_O]，131.04[C_9_H_7_O]； | C |
| 49 | 14.45 | 3,5-Dicaffeoylquinic acid | 516.12678 | -H | C_25_H_24_O_12_ | 516.1274 | 515.1202 | 0.7 | 1.3 | 1494279 | 93.03[C_6_H_5_O]，  135.04[C_8_H_7_O_2_]； | B |
| 50 | 14.8 | Ningposide B | 382.12638 | -H | C_18_H_22_O_9_ | 382.1279 | 381.1207 | 1.6 | 4.1 | 920 | 161.02[C_9_H_5_O_3_]，179.03[C_9_H_7_O_4_]； | A |
| 51 | 14.96 | Bergapten | 216.0425 | +H | C_12_H_8_O_4_ | 216.04226 | 217.0498 | 0.2 | 1.1 | 4134 | 151.04[C_8_H_7_O_3_]，165.05[C_9_H_9_O_3_]； | D |
| 52 | 15.28 | Angoroside C | 784.27898 | -H | C_36_H_48_O_19_ | 784.2799 | 783.2726 | 0.9 | 1.1 | 928639 | 175.03[C_10_H_7_O_3_]，509.16[C_24_H_29_O_12_]； | A |
| 53 | 15.45 | Caryophyllene oxide | 220.183 | +H | C_15_H_24_O | 220.18272 | 221.1903 | 0.3 | 1.4 | 1789 | 107.04[C_7_H_7_O]，123.11[C_9_H_15_]； | B |
| 54 | 15.53 | Formononetin | 268.07356 | -H | C_16_H_12_O_4_ | 268.0743 | 267.067 | 0.7 | 2.7 | 24097 | 132.02[C_8_H_4_O_2_]，251.03[C_15_H_7_O_4_]； | D |
| 55 | 15.6 | Isoliquiritin | 418.12638 | -H | C_21_H_22_O_9_ | 418.1271 | 417.1198 | 0.7 | 1.7 | 249632 | 225.06[C_15_H_11_O_4_]，135.00[C_7_H_3_O_3_]； | D |
| 56 | 16.01 | Licochalcone B | 286.0848 | +H | C_16_H_14_O_5_ | 286.08412 | 287.0921 | 0.7 | 2.3 | 183901 | 163.03[C_9_H_7_O_3_]，121.02[C_7_H_5_O_2_]； | D |
| 57 | 16.63 | Sebacic acid | 202.12051 | -H | C_10_H_18_O_4_ | 202.1207 | 201.1134 | 0.2 | 1 | 2777 | 139.11[C_9_H_15_O]，183.10[C_10_H_15_O_3_]； | B |
| 58 | 16.69 | Cistanoside D | 652.23672 | -H | C_31_H_40_O_15_ | 652.2379 | 651.2306 | 1.2 | 1.8 | 15360 | 175.04[C_10_H_7_O_3_]，565.17[C_30_H_29_O_11_]； | A |
| 59 | 17.05 | Luteolin | 286.04774 | -H | C_15_H_10_O_6_ | 286.0486 | 285.0413 | 0.8 | 2.9 | 5449 | 133.02[C_8_H_5_O_2_]，179.03[C_9_H_7_O_4_]； | B |
| 60 | 17.55 | 4,5-O-Dicaffeoylquinic acid | 516.1278 | +H | [C_28_H_26_O_15_](https://pubchem.ncbi.nlm.nih.gov/#query=C28H26O15) | 516.12678 | 517.1351 | 1 | 1.9 | 169063 | 137.05[C_8_H_9_O_2_]，188.07[C_8_H_12_O_5_]； | B |
| 61 | 17.74 | (E)-Aldosecologanin | 742.2753 | +H | [C_34_H_46_O_19_](https://pubchem.ncbi.nlm.nih.gov/#query=C34H46O19) | 742.26841 | 743.2826 | 6.9 | 9.2 | 3542 | 151.03[C_8_H_7_O_3_]，240.06[C_11_H_12_O_6_]； | B |
| 62 | 17.83 | Scropolioside A | 752.2528 | +H | [C_35_H_44_O_18_](https://pubchem.ncbi.nlm.nih.gov/#query=C35H44O18) | 752.25276 | 753.2601 | 0 | 0 | 10130 | 149.06[C_9_H_9_O_2_]，517.16[C_26_H_29_O_11_]； | A |
| 63 | 17.95 | Harpagoside | 494.17881 | -H | C_24_H_30_O_11_ | 494.1797 | 493.1725 | 0.9 | 1.9 | 157719 | 147.04[C_9_H_7_O_2_]，345.12[C_15_H_21_O_9_]； | A |
| 64 | 18.46 | Deoxyglabrolide | 454.3445 | +H | C_30_H_46_O_3_ | 454.3447 | 455.3518 | -0.2 | -0.4 | 157062 | 437.34[C_30_H_45_O_2_]； | D |
| 65 | 19.68 | Isoglabrolide | 468.3241 | +H | C_30_H_44_O_4_ | 468.32396 | 469.3313 | 0.1 | 0.2 | 15476 | 409.34[C_29_H_45_O]，437.34[C_30_H_45_O_2_]； | D |
| 66 | 20.11 | E-Ligustilide | 190.0997 | +H | [C_12_H_14_O_2_](https://pubchem.ncbi.nlm.nih.gov/#query=C12H14O2) | 190.09938 | 191.107 | 0.4 | 1.9 | 63022 | 121.02[C_7_H_5_O_2_]，147.04[C_9_H_7_O_2_]； | C |
| 67 | 20.46 | 2-Caren-4-ol | 152.1205 | +H | [C_10_H_16_O](https://pubchem.ncbi.nlm.nih.gov/#query=C10H16O) | 152.12012 | 153.1278 | 0.4 | 2.7 | 1839 | 93.07[C_7_H_9_]，135.11[C_10_H_15_]； | B |
| 68 | 20.73 | Glycyrrhizic acid | 822.4037 | +H | C_42_H_62_O_16_ | 822.40379 | 823.4109 | -0.1 | -0.1 | 5571841 | 471.34[C_30_H_47_O_4_]，647.37[C_36_H_55_O_10_]； | D |
| 68 | 20.78 | Glycyrrhizic acid | 822.40379 | -H | C_42_H_62_O_16_ | 822.4041 | 821.3969 | 0.4 | 0.4 | 4523600 | 351.05[C_12_H_15_O_12_]，759.39[C_41_H_59_O_13_]； | D |
| 69 | 20.79 | Cistanoside F | 488.1497 | +H | C_21_H_28_O_13_ | 488.15299 | 489.1569 | -3.3 | -6.8 | 7713 | 431.18[C_20_H_31_O_10_]； | A |
| 70 | 21.01 | Ursolic acid | 456.3589 | +H | C_30_H_48_O_3_ | 456.36035 | 457.3662 | -1.4 | -3.2 | 18814 | 137.05[C_8_H_9_O_2_]，318.30[C_22_H_38_O],  439.35[C_30_H_47_O_2_]； | C |
| 71 | 21.48 | Cnidilide | 194.1304 | +H | C_12_H_18_O_2_ | 194.13068 | 195.1377 | -0.3 | -1.4 | 3272 | 105.07[C_8_H_9_]，149.05[C_9_H_9_O_2_]； | C |
| 72 | 21.71 | Licobenzofuran | 354.1467 | +H | C_21_H_22_O_5_ | 354.14672 | 355.154 | 0 | -0.1 | 31398 | 147.11[C_11_H_15_]，299.09[C_17_H_15_O_5_]； | D |
| 73 | 21.74 | Neryl acetate | 196.14633 | -H | C_12_H_20_O_2_ | 196.146 | 195.1388 | -0.3 | -1.5 | 83 | 109.03[C_6_H_5_O_2_]，181.12[C_11_H_17_O_2_]； | B |
| 74 | 21.93 | Quercetin-3',4',7-trimethyl ether | 328.09469 | -H | C_18_H_16_O_6_ | 328.0937 | 327.0864 | -1 | -3.1 | 90 | 165.02[C_8_H_5_O_4_]； | B |
| 75 | 22.16 | Licoricone | 382.14164 | -H | C_22_H_22_O_6_ | 382.1428 | 381.1355 | 1.1 | 3 | 12642 | 323.05[C_18_H_11_O_6_]，351.08[C_18_H_11_O_6_]； | D |
| 76 | 22.43 | 24-Hydroxyglycyrrhetic acid | 500.3509 | +H | [C_30_H_46_O_5_](https://pubchem.ncbi.nlm.nih.gov/#query=C30H46O5) | 500.35017 | 501.3582 | 0.7 | 1.4 | 5736 | 219.17[C_15_H_23_O]，402.32[C_26_H_42_O_3_]； | D |
| 77 | 22.89 | Sugiol | 300.2089 | +H | C_20_H_28_O_2_ | 300.20893 | 301.2162 | 0 | 0 | 2458 | 95.08[C_7_H_11_]，175.11[C_12_H_15_O]； | A |
| 78 | 22.97 | Methyl glycyrrhetate | 484.352 | +H | C_31_H_48_O_4_ | 484.35526 | 485.3593 | -3.2 | -6.6 | 2102 | 135.11[C_10_H_15_]，247.24[C_18_H_31_]； | D |
| 79 | 23.58 | Alloocimene | 136.1255 | +H | C_10_H_16_ | 136.1252 | 137.1328 | 0.3 | 2.5 | 3336 | 95.08[C_7_H_11_]，123.11[C_9_H_15_]； | C |

*Scrophulariae Ningpoensis* (A)；Flos *Lonicerae Japonicae* (B)；Radix *Scrophulariae Ningpoensis* (C); Radix *Glycyrrhizae Uralensis* (D)
